# *Sphagnum cuspidatulum* extract prevents acute kidney injury induced by high-fat diet and streptozotocin via alleviation of oxidative stress and apoptosis in pre-diabetic rats

**DOI:** 10.3389/fphar.2024.1464463

**Published:** 2024-10-22

**Authors:** Pongrapee Laorodphun, Sutheera Chaisen, Sarocha Amattat, Pornchita Maphet, Narin Printrakul, Hataichanok Pandith, Aussara Panya, Burit Kongmali, Myat Theingi Swe, Phatchawan Arjinajarn

**Affiliations:** ^1^ Department of Biology, Faculty of Science, Chiang Mai University, Chiang Mai, Thailand; ^2^ Interdisciplinary Program in Biotechnology, Faculty of Graduate School, Chiang Mai University, Chiang Mai, Thailand; ^3^ Department of Physiology, University of Medicine 2, Yangon, Myanmar

**Keywords:** peat moss, phenolic compounds, pre-diabetic, renal injury, obesity

## Abstract

**Context:**

Obesity and pre-diabetes are associated with renal dysfunction via elevated oxidative stress. Peat moss, or *Sphagnum cuspidatulum* Müll. Hal., Sphagnaceae (SC), are rich in phenolic compounds that enhance antioxidant activity.

**Objective:**

SC might show beneficial effects in pre-diabetes-associated renal dysfunction.

**Materials and methods:**

Male Wistar rats, after 4 weeks on a high-fat diet, received low-dose streptozotocin to induce pre-diabetes. Then, the pre-diabetic rats were randomly divided into 4 groups: untreated pre-diabetic rats (P-DM), pre-diabetic rats treated with SC 50 or 100 mg/kg/day (P-DM50 or P-DM100), and pre-diabetic rats treated with metformin 100 mg/kg/day (MET). The drugs were fed by gavage for 4 weeks.

**Results:**

Treatment with SC100 dramatically lowered serum creatinine (S.Cr.), blood urea nitrogen (BUN), and augmented creatinine clearance in pre-diabetic rats. Additionally, SC100 significantly decreased the malondialdehyde level. Furthermore, pre-diabetic rats treated with SC100 significantly upregulated the expression of nuclear factor erythroid 2-related factor 2 (Nrf2) and its downstream mediators, with downregulated apoptotic markers.

**Discussion and conclusion:**

Our findings provide a scientific basis for the clinical application of SC and a new strategy for the prevention of nephrotoxicity and other kidney disease in the future.

## Introduction

Obesity is considered a worldwide health burden as it increases the risk of various chronic diseases, including type 2 diabetes (T2D), cardiovascular disease, liver disease, and chronic kidney disease (CKD) (Ahmed et al., 2021). CKD increases the risk of end-stage renal function if left untreated. From a metabolic perspective, the imbalance between the body’s energy intake and expenditure is one of the primary variables contributing to obesity. Excessive consumption of a high-fat diet, obesity, and obesity-related kidney diseases are strongly associated with systemic oxidative stress, both in humans and animals (Savini et al., 2013). Reactive oxygen species (ROS) are overproduced in obesity and have a crucial role in the development of T2D and associated consequences (Jaikumkao et al., 2018). Elevated levels of malonaldehyde (MDA) have been seen in patients with obesity, demonstrating a significant association with body mass index (BMI), body fat percentage, low-density lipoprotein (LDL) levels, and triglyceride (TG) levels (Islayem et al., 2022).

Diabetic nephropathy and diabetes associated renal dysfunction are a common complication in T2D. The glomerular filtration barrier, a three-part system consisting of fenestrated endothelial cells, the glomerular basement membrane, and podocytes, deteriorates in response to oxidative stress. Additionally, nephrin, a protein biomarker that reflects podocyte function, becomes downregulated (Drummond and Mauer, 2002; Ponchiardi et al., 2013) whereas excessive energy intake leads to renal injury by promoting abnormal lipid accumulation and triggering epigenetic activation of pro-lipogenic and pro-fibrotic signaling pathways within the renal tissue (Chen et al., 2019). Furthermore, epithelial mesenchymal transition (EMT) and endothelial mesenchymal transition (EndMT) have been well documented to play a crucial role in diabetic renal fibrosis (Liu et al., 2022). Several responsible signaling pathway, i.e., endothelial fibroblast growth factor receptor 1 (FGFR1) (Srivastava et al., 2020; Wu et al., 2018), endothelial Sirtuin3 (SIRT3)-mediated signaling mechanism (Quan et al., 2020), dipeptidyl peptidase-4 (DPP-4)-mediated signaling mechanism (Daza-Arnedo et al., 2021; Hsu et al., 2022), glucocorticoid receptor signaling (Srivastava et al., 2021a) therefore becomes as key target to inhibit renal fibrosis and related kidney injury. Protein kinase C (PKC) plays a pivotal role in the process of signal transduction and the regulation of gene expression (Singh et al., 2017). Its involvement in oxidative stress has been reported recently. In the presence of oxidative stress, the PKC becomes excessively active, leading to the translocation of nuclear factor erythroid 2-related factor 2 (Nrf-2). Nrf-2 subsequently triggers the activation of several antioxidant enzymes, including glutamate-cysteine ligase (GCLC), superoxide dismutase (SOD), and heme oxygenase-1 (HO-1) (Ma, 2013). Moreover, previous studies have indicated that increased production of reactive ROS within cells might exert an impact on the B-cell lymphoma 2 (Bcl-2) protein family and the caspase cascade, hence leading to cellular apoptosis (Arjinajarn et al., 2016).

The landscape of diabetes management is rapidly evolving as traditional treatments have not fully addressed the underlying causes of the disease and are often associated with significant side effects. Currently, the treatment of T2D includes insulin injections and oral hypoglycemic agents, which play key roles in managing the disease but are also associated with adverse effects. Despite advances, several challenges remain, including optimizing treatments for effective glycemic, lipid, and blood pressure control, and addressing safety. Specifically, certain treatments have been employed to manage T2D and mitigate associated kidney injury. Therapies such as SIRT3 activators, glycolysis inhibitors, DPP-4 inhibitors (e.g., linagliptin), and the peptide AcSDKP are increasingly utilized in both clinical practice and research to prevent kidney damage. For example, DPP-4 inhibitors, such as vildagliptin, are approved for the treatment of type 2 diabetes. By inhibiting DPP-4, vildagliptin increases and prolongs GLP-1 levels, enhancing insulin secretion and reducing blood glucose levels (D’Alessio et al., 2009). Previous studies reported that vildagliptin treated in type 1 and 2 diabetic rats showed significant attenuated blood glucose, improved lipid profiles, and also decreased oxidative stress (Ali et al., 2015). A recent meta-analysis reported the beneficial effect of DPP-4 inhibitors including vildagliptin and alogliptin on total cholesterol and triglyceride levels compared to placebo (Monami et al., 2012).

In recent years, natural compounds have become a new strategy to counteract metabolic diseases, and plant-extracted phenolic compounds have become a central position in nutrition research. *Sphagnum cuspidatulum* Müll. Hal., Sphagnaceae (SC) extract has been used as a folk medicine to treat certain diseases in several Asian countries, including Thailand. SC is a semi-aquatic moss that can be found in Aug Ka Doi Inthanon, Chiang Mai, Thailand. It contains many flavonoids and phenolic compounds, which have been traditionally used to treat stroke and chest pain (Chen and Zhang, 2021). Apparently, SC contained high levels of phenolic compounds that can act as an anticancer agent by ameliorating oxidative stress and inhibiting cell division (Sharifi-Rad et al., 2023). Numerous studies have shown that phenolic compounds can prevent and improve several chronic illnesses as they have anti-atherosclerotic, antitumor, antidiabetic, anti-inflammatory, and anticancer properties (Rahman et al., 2021). However, the effects of SC extract on obesity and pre-diabetic-associated renal dysfunction have not been reported yet. Therefore, the present study is designed to investigate the effects and related underlying mechanisms of SC extract on the renal function of obese pre-diabetic rats.

## Materials and methods

### Sample collection and preparation

Gametophores of *Sphagnum cuspidatulum* were harvested on 5 September 2021 from Ang Ka swamp under Rhododendron (Ericaceae) at about 2,550 m elevation near the summit of Doi Inthanon National Park (Permit number: TS0907.4/29044), Chiang Mai Province, Northern Thailand, 18° 35′ 20″ N and 98° 29′ 10″ E. The voucher specimens (Printarakul 05092021_5) were housed in the CMUB herbarium, Biology Department, Faculty of Science, Chiang Mai University, Thailand. The sample was extracted using absolute methanol (AR grade, RCI Labscan, Bangkok, Thailand) for 24 h at room temperature three times, at a ratio of 1:10 w/v (Salih et al., 2021). The solution was purified prior to being used in combination with Whatman No. 1 (Cytiva, Marlborough, Massachusetts, United States) filter paper. Using a rotatory evaporator, the solvent was eliminated, and it was then dried in a fume hood. Then, until use, the crude extract was kept in an amber bottle at −20°C.

### Preparation of caffeine from *Sphagnum cuspidatulum*

To prepare the peat moss sample exhibiting the highest quantity of caffeine, the procedure performed by Antioxidant Activities (AAs) was utilized. The first step was an optimization process to determine the most effective extraction conditions for caffeine from peat moss. Once these ideal conditions were established, they were then utilized in the subsequent experiments. The extraction method was carried out over a period of 3 h at room temperature, with constant agitation at a rate of 150 rpm. Following that, the mixture was filtered and evaporated under decreased pressure at a temperature of 40°C. The procedure led to the extraction of the aqueous residual fraction from the whole-stem peat moss. Subsequently, the fraction underwent evaporation to remove all liquid content and was subjected to fractionation using solid-phase extraction. This process involved utilizing a 75 × 8 cm Amberlite XAD7HP column. Subsequently, the fraction was subjected to defatting by partitioning with hexane, leading to the formation of a fraction enriched in caffeine.

### Determination of total polyphenolic compounds and identification of bioactive compounds in crude extract

The Folin-Ciocalteu method, was used for the quantitative measurement of polyphenolic chemicals. The crude extract was solubilized in a solution consisting of 80% methanol. Subsequently, a mixture was prepared by combining 60 µL of the crude extract with 2.5 mL of Folin-Ciocalteu’s phenol reagent. Following a period of incubation in the absence of light for a duration of 10 min, a 2 mL volume of sodium carbonate (Na_2_CO_3_) solution with a concentration of 7.5% was introduced to the solution. Subsequently, the solution was subjected to incubation at a temperature of 50°C for a duration of 15 min, until the completion of the reaction. The measurement of absorbance was determined using a spectrophotometer set at a wavelength of 760 nm. The gallic acid was produced as a standard reagent at concentrations of 25, 50, 100, 200, and 400 μg/mL.

The bioactive chemicals were analyzed using the gas chromatography-mass spectrometry (GC-MS) technique, which involved the utilization of an Agilent Technologies 7890b gas chromatograph and an MSD 5977B mass spectrometer. Initially, a 1 µL sample dissolved in 80% methanol was introduced into a GC-MS system. The injection was performed using the split (10:1) inlet mode. The sample was then separated using a non-polar phenyl arylene polymer column (DB-5MS, 30 m × 0.25 mm, 0.5 μm film thickness) with a temperature gradient. The gradient started at 0°C and increased at a rate of 4°C/min until reaching 100°C, where it was held for 10 min. Subsequently, the temperature was further increased at a rate of 4°C/min from 100°C to 270°C and held for 20 min. Finally, the temperature was raised from 270°C to 280°C at a rate of 4°C/min and held for 20 min. Helium gas was used as the carrier gas at a flow rate of 1.9 mL/min. The identification of bioactive chemicals was conducted using a mass spectrometry (MS) instrument operating in electron impact mode. The mass range analyzed was m/z 25–700, and an electron energy of 70 eV was utilized. The mass spectra were discovered using a comparative analysis with the Wiley and NIST libraries.

The samples that underwent sialylation were introduced into a GC-MS system consisting of an MSD 5977B mass spectrometer and a 7890b gas chromatograph, both manufactured by Agilent Technologies in Shanghai, China. The configuration of the system involved operating in the Electron Impact (EI) mode, utilizing a mass range spanning from m/z 25 to 700 with an electron energy of 70 electron volts (eV). The experiment utilized a capillary column DB-5MS with dimensions of 30 m × 0.25 mm (i.d.) and a coating substance with a film thickness of 0.5 μm. Both the detector and injector were set to a temperature of 280°C. GC was performed in splitless mode with a 1-min splitless duration. The temperature protocol involved a gradual increase in temperature from 50°C to 100°C at a rate of 4°C per min. This temperature was then maintained for a duration of 10 min. Subsequently, the temperature was further increased from 100°C to 270°C at the same rate of 4°C per min and held at 270°C for a period of 20 min. Finally, the temperature was raised from 270°C to 280°C at a rate of 4°C per min and maintained at 280°C for an additional 20 min. A post-run duration of 10 min at a temperature of 50°C was found to be enough for the second injection. The carrier gas, helium, maintained a constant flow rate of 1.9 mL/min. Compounds were identified by means of comparing their retention periods with those of authentic compounds and utilizing the spectrum information available in the Wiley and NIST libraries. The process of decision-making was replicated.

### Animals

The male Wistar rats, with an average weight ranging from 180 to 200 g, were obtained from Nomura Siam International, a supplier based in Bangkok, Thailand. The animals require 7 days to adapt to their unusual surroundings. The rats utilized as experimental subjects were housed in a controlled environment with certain conditions. These conditions included a constant temperature of 25°C, a humidity level of 55%, and a 12 h light/dark cycle. During the acclimatization phase, the rats were given full access to a regular pellet diet and distilled water. The Laboratory Animal Center at Chiang Mai University in Chiang Mai, Thailand, has provided approval (Permit Number: 2565/RT-0025) for the animal facilities and procedures used during this work.

### Experimental design

The rats were randomly allocated to two dietary groups: the normal diet (ND) group and the high-fat diet (HFD) group. In the normal diet group, a total of 16 rats were administered the ordinary chow diet (C.P. Mice Feed Food No. 082), which included 19.77% of calories derived from fat. The rats in the high-fat diet groups (n = 32) were administered a food with a high-fat content, comprising 57.60% of their calorie intake. Prior to inducing diabetic impairment, rats were maintained on a high-fat diet for a duration of 4 weeks. To induce T2D, rats were administered a high-fat meal and afterwards received an intraperitoneal injection of nicotinamide (120 mg/kg), followed by a low dosage of streptozotocin (STZ) (40 mg/kg). In contrast, the control group of animals received citrate buffer instead. The rats that were fed with ND were divided into two groups consisting of 8 rats each. One group received a vehicle, while the other group received a SC extract (ND100) at a dosage of 100 mg/kg/day. This division was done after conducting an oral glucose tolerance test (OGTT) to confirm the validity of the model. The rats that were given a high-fat diet were then divided into four groups consisting of 8 rats each. These groups were as follows: pre-diabetic rats treated with normal saline (NSS) (referred to as P-DM), pre-diabetic rats treated with an extract from SC (referred to as P-DM50; administered at a dosage of 50 mg/kg/day), pre-diabetic rats treated with an extract from SC (referred to as P-DM100; administered at a dosage of 100 mg/kg/day), and pre-diabetic rats treated with metformin (referred to as MET). The dosage of SC extract was chosen based on a previous study (Afolabi et al., 2023) in which the caffeine levels used were either equal to or lower than those in our study. The study demonstrated that such caffeine dosages provided beneficial effects without causing any toxicity effect in a rat model of aluminium chloride (AlCl3) induced nephrotoxicity. Oral administration of SC extract and metformin was conducted for a duration of 4 weeks using a gavage technique, after the immediate dissolution in NSS.

The energy intake was calculated using equations:

$$\text{Energy intake of ND }\left(\text{Kcal}/\text{day}\right)=\text{food intake}\left(g/\text{day}\right)\times \text{constant }\text{of standard chow }\left(4.2\text{ Kcal}/g\right)$$

$$\text{Energy }\text{intake of HF }\left(\text{Kcal}/\text{day}\right)=\text{food intake}\left(g/\text{day}\right)\times \text{constant of }\text{high}-\text{fat diet }\left(5.35\text{ Kcal}/g\right)$$

### Oral glucose tolerance test (OGTT)

The administration of the OGTT took place in the fourth and eighth weeks. The rats were subjected to gavage administration of a glucose solution (2 g/kg) after a period of fasting overnight. Blood samples were obtained from the distal end of the tail at various time intervals, encompassing the pre-glucose loading phase (0 min) as well as the post-glucose injection periods of 30, 60, and 120 min, during which the subjects were anesthetized. Colorimetric test kits (Erba Diagnostics Mannheim GmbH, Mannheim, Germany) were utilized to measure blood glucose levels. Following that, the evaluation of impaired glucose tolerance was performed using the trapezoidal formula, which entailed calculating the area under the curve for glucose levels during the OGTT, generally known as TAUCg.

### Blood and renal tissue sampling

The collection of 24 h urine samples was conducted before each OGTT trial, utilizing individual metabolic cages. The animals were subjected to euthanasia by the administration of an excessive amount of the anesthetic 2% isoflurane (Hawkley et al., 2024), and thereafter blood samples were obtained from the abdominal aorta. The serum and plasma samples underwent separation prior to use and were then kept at a temperature of −20°C. The kidneys were promptly removed, devoid of their capsules, and assessed in terms of their weight. For histological study, the kidney was partitioned into two segments. The initial portion of the experiment involved submerging the sample in a 4% solution of recently made paraformaldehyde at a pH level of 7.4 to achieve fixation. The last portion of the experiment involved meticulous partitioning to separate the renal cortex, which was then preserved at a temperature of −80°C. The preserved cortical tissue will be employed in future research that encompasses Western blotting as well as the examination of tissue MDA levels, glutathione (GSH), and reducing glutathione (GSSG) content.

### Blood parameter assessment

A colorimetric test kit was used to quantify the concentrations of triglyceride, cholesterol, and fasting plasma glucose (Erba Diagnostics Mannheim GmbH, Mannheim, Germany). Plasma HDL was determined by using an automated chemical analyzer (Sysmex BX-3010, Gobe, Japan). The LDL was calculated using the standard formula.

$$\text{LDL}-c=\text{total cholesterol }–\text{ HDL }–\;\left(\text{triglyceride}/5\right)$$

### Renal function assessment

Serum creatinine and blood urea nitrogen (BUN) levels were measured using an automated analyzer (Sysmex BX-3010, Gobe, Japan). The creatinine clearance was calculated using the standard formula.

C=UV/P

where C was creatinine clearance, U was the urine concentration of creatinine in mg/dL, V was the urine flow rate per min in mL/min, and P was the plasma concentration of creatinine in mg/dL.

### Determination of renal oxidative stress

A thiobarbituric acid reactive substances (TBARS) test kit was used to measure the amount of MDA in the renal cortical tissue (Cayman Chemical Company, Ann Arbor, Michigan, United States). A commercial assay kit was used to measure the level of GSH/GSSG in the renal cortex (EGTT-100, Bioassay Systems, Hayward, California, United States) according to the manufacturer’s instructions.

### Western blotting

Renal cortical tissues were subjected to the normal procedure for Western blotting. Kidney cortex samples were cut into slices and mixed with mammalian lysis solution from Sigma-Aldrich (Sigma-Aldrich, St. Louis, Missouri, United States) to get whole-cell kidney lysate that also contained a protease inhibitor (Roche, Indianapolis, Indiana, United States). This procedure was conducted in a centrifuge at 10,000 *g* for 15 min at 4°C. Equal volumes of protein samples were loaded into 10 or 12% of sodium dodecyl sulfate-polyacrylamide gels (SDS-PAGE), then electrophoresed and transferred to 0.2 μm polyvinylidene fluoride (PVDF) membranes (Bio-Rad, Pennsylvania, United States). Following this, membranes were blocked with 5% BSA in PBS or TBS for an hour at room temperature. The primary antibodies used were anti-Bax (Cat# ab182733), Bcl-2 (Cat# ab194583), HO-1 (Cat# ab13248), GCLC (Cat# 13475), and nephrin (Cat# ab216341), antibodies originated from Abcam (Abcam, Cambridge, United Kingdom). The antibodies targeting PKC-α (Cat# sc-208) and Nrf-2 (Cat# 33649) were acquired from Santa Cruz Biotechnology (Santa Cruz, California, United States). The SOD2 (Cat# 13149), α-SMA (Cat# 19245), Cytochrome C (Cat# 4272) and, TGF-β (Cat# 3711) antibodies were purchased from Cell Signaling Technology (Cell Signaling Technology, Massachusetts, United States). The membranes were probed with these primary antibodies at 4°C overnight. After that, membranes were rinsed and incubated with an HRP-conjugated anti-mouse or anti-rabbit secondary antibody (Amersham, Illinois, United States) for an hour at room temperature. Thereafter, proteins were detected by enhanced chemiluminescence agent (ECL) and Chemi Doc imaging systems (Image Quant LAS500, GE Healthcare Limited, Buckinghamshire, United Kingdom). The densities of the bands were analyzed using ImageJ software (National Institutes of Health, Bethesda, Maryland, United States). As a loading control, the anti-β-actin (Cat# 5125) antibody was utilized.

### Histological examination

The kidneys were prepared for preservation by immersing them in a 4% solution of fresh paraformaldehyde (pH 7.4) for a duration of 24 h. Subsequently, the preserved kidneys were embedded in paraffin to perform histological analyses. The kidneys were sectioned into slices with a thickness of 5 micrometers. Subsequently, these slices were subjected to counterstaining using hematoxylin and eosin (H&E) and examined using an Olympus light microscope (Olympus America Inc., New York, United States). (40 × magnification) by an observer, who was blinded to the treatment groups. The evaluation of the kidney injury score was conducted based on the subsequent semi-quantitative scoring system (Laorodphun et al., 2021). The staining techniques utilized in this study were periodic acid-Schiff (PAS) and Masson’s trichrome. These stains were applied to kidney slides to assess the presence of glycogen buildup, determine the glomerulosclerotic index, and examine the deposition of collagenous connective tissue fibers.

### Statistical analysis

The statistical analysis was conducted with GraphPad Prism version 8 (Graph Pad Software, La Jolla, California, United States). The data were presented using the mean ± standard error of the mean (SEM) format. The statistical methods applied in this study encompassed the application of an independent sample t-test was used to compare the differences between two groups and analysis of variance (ANOVA) with Fisher’s Least Significant Difference (LSD) *post hoc* tests. These approaches were performed to evaluate the statistical significance of the observed disparities. Statistical significance was determined using a significance threshold of *p* < 0.05.

## Results

### Bioactive compounds in crude extract

**FIGURE 1:**
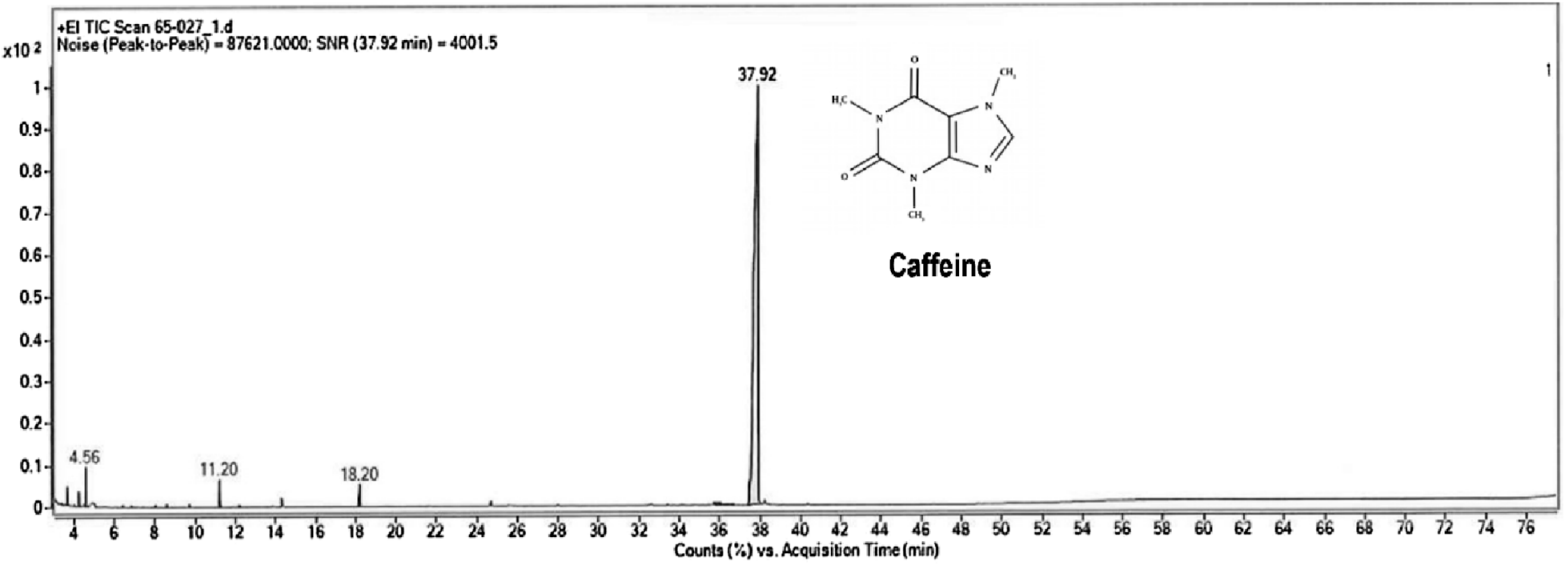
Gas chromatogram with mass-spectrometric detection of the total lipid extract of *Sphagnum cuspidatulum* (SC).

The crude extract of *Sphagnum cuspidatulum* was characterized to determine the total phenolic content and identified the bioactive compounds. The result showed that *S. cuspidatulum* exhibited a high concentration of polyphenolic chemicals, with a measured content of 18.1083 mg GAE/mL using the Folin-Ciocalteu technique. The gas chromatography/mass spectrometry (GC-MS) approach successfully detected six substances, namely, propanoic acid, 2-oxo-methylester, 3,4-dimethyl-3-pyrrolin-2-one, 2-hydroxymethyl-2-methyl-pyrrolidine-1-carboxaldehyde, 1,1,3,3-tetramethy-1,3-disilaphenalane, and 1,3,7-trimethylpurine-2,6-dione, which is also known as caffeine (Figure 1). The primary bioactive molecule, caffeine, was shown to have the greatest peak height on the chromatogram, as evidenced by its detection at a retention time of 37.92 min.

### The effect of HF diet and low does STZ on metabolic and renal parameters at 4th week

Table 1 displays the metabolic parameters observed at the fourth week. Before the initiation of the SC extract therapy, the HF groups exhibited substantially elevated levels of body weight, energy intake, plasma glucose, total area under the curve for glucose (TAUCg), plasma cholesterol, and plasma LDL compared to the ND (*p* < 0.05) (Table 1). Significant reductions in HDL levels, urine creatinine and creatinine clearance were seen in HF group in comparison to the ND group (*p* < 0.05). It was also observed that the HF groups demonstrated significantly increased levels of serum creatinine and urine protein than the ND group (*p* < 0.05). However, there was no observable disparity among the experimental groups with regards to the blood urea nitrogen (BUN) level and urine volume. These findings indicate that the administration of a high-fat diet alongside a moderate dose of streptozotocin (STZ) resulted in the manifestation of pre-diabetic condition and a deterioration in renal function.

**TABLE 1:** Metabolic parameters in male Wistar rats at 4 weeks of high-fat diet consumption (Before treatment).

	ND	HF
Body weight (g)	441.42 ± 4.98	482.08 ± 5.18*
Energy intake (Kcal/day)	88.84 ± 2.73	108.20 ± 2.47*
Fasting blood glucose (mg/dL)	113.38 ± 1.58	145.12 ± 3.41*
TAUCg (mg/dL x min x 10^3^)	16.57 ± 0.20	20.28 ± 0.25*
Cholesterol (mg/dL)	55.20 ± 2.04	114.47 ± 3.14*
Triglyceride (mg/dL)	18.70 ± 1.38	24.50 ± 1.61*
HDL (mg/dL)	48.10 ± 1.06	33.50 ± 0.57*
LDL (mg/dL)	16.34 ± 2.51	66.64 ± 3.37*
S.Cr (mg/dL)	0.550 ± 0.005	0.617 ± 0.005*
BUN (mg/dL)	12.91 ± 0.50	15.00 ± 0.77
U.Cr (mg/dL)	88.73 ± 2.41	68.49 ± 4.85*
Creatinine clearance (mL/min)	1.85 ± 0.07	1.21 ± 0.08*
U.Protein (g/dL)	111.79 ± 4.11	170.83 ± 6.06*
24-h U.Volume (mL/day)	19.60 ± 0.72	19.40 ± 1.14

Data are presented as mean ± standard error of mean (SEM) ND, normal diet rats treated with vehicle, n = 10 rats/group (t-test, *p* < 0.05). HF, high-fat diet rats, n = 20 rats/group (t-test, *p* < 0.05).

### Effect of SC extract on metabolic status

After 8 weeks, it was observed that the P-DM group exhibited notably elevated levels of body weight, energy intake, fasting plasma glucose, total area under the curve for glucose (TAUCg), plasma cholesterol, plasma triglyceride, low-density lipoprotein (LDL), and visceral fat weight. The statistical significance (*p* < 0.05) was seen in comparison to the ND and ND100 groups (Table 2). When P-DM was contrasted with the ND and ND100 groups, HDL was lower in P-DM (*p* < 0.05). Interestingly, the P-DM100 rats that received SC extract at doses of 100 mg/kg/day showed a significant improvement in metabolic parameters as measured by fasting plasma glucose, TAUCg, and HDL levels. Additionally, in P-DM100 groups, visceral fat weight, plasma cholesterol, and plasma LDL exhibited a declining tendency. Rats that were fed a high-fat diet exhibited reduced 24 h urine volume and creatinine clearance, along with increased kidney weight and elevated levels of serum creatinine and BUN in comparison to ND and ND100 (*p* < 0.05). After receiving SC treatment, serum creatinine was considerably lower in the P-DM50 and P-DM100 groups compared to the P-DM group. In contrast, when compared to P-DM, the P-DM100 group showed a considerably greater increase in creatinine clearance (*p* < 0.05) (Table 2). The 24 h urine volume was improved in the SC treated groups when compared with pre-diabetic rats (*p* < 0.05).

**TABLE 2:** Metabolic parameters and renal functions in male Wistar rats at 8 weeks (After treatment).

	ND	ND100	P-DM	P-DM50	P-DM100	MET
Body weight (g)	474.53 ± 30.45	473.71 ± 5.09	474.12 ± 25.35*	537.26 ± 16.96*	521.02 ± 17.03	551.01 ± 15.23*
Energy intake (Kcal/day)	78.13 ± 4.46*	74.54 ± 2.52	122.29 ± 0.84*	112.50 ± 2.13*	104.90 ± 1.74*	116.40 ± 0.91*
Fasting blood glucose (mg/dL)	101.2 ± 1.69	99.0 ± 1.52	188.6 ± 31.56*	133.0 ± 1.22^†^	126.2 ± 2.29^†^	127.46 ± 4.04^†^
TAUCg (mg/dL × min × 10^3^)	15.25 ± 0.15	15.29 ± 0.24	21.17 ± 0.73*	18.79 ± 0.55*^,†^	17.29 ± 0.46*^,†,‡^	16.79 ± 0.64*^,†,‡^
Cholesterol (mg/dL)	63.03 ± 5.84	60.28 ± 9.97	122.52 ± 19.02*	98.51 ± 9.06*	85.29 ± 9.54^†^	60.72 ± 4.40^†^
Triglyceride (mg/dL)	24.92 ± 1.78	27.4 ± 1.48	66.63 ± 4.49*	54.51 ± 5.28*	49.16 ± 6.26*	47.92 ± 11.43*^,†^
HDL (mg/dL)	46.0 ± 2.41	49.4 ± 2.96	39.4 ± 1.77	45.4 ± 2.75	53.4 ± 6.24^†^	42.8 ± 3.60^†^
LDL (mg/dL)	22.21 ± 5.09	19.30 ± 3.04	66.34 ± 14.62*	38.05 ± 8.06^†^	32.35 ± 12.66^†^	19.49 ± 1.21^†^
S.Cr (mg/dL)	0.65 ± 0.002	0.67 ± 0.002	0.80 ± 0.03*	0.73 ± 0.02^†^	0.71 ± 0.03*^,†^	0.66 ± 0.01^†,‡^
BUN (mg/dL)	13.46 ± 0.56	14.66 ± 1.14	26.54 ± 2.25*	13.62 ± 0.89^†^	14.02 ± 2.16^†^	13.92 ± 1.0^†^
U.Cr (mg/dL)	113.59 ± 4.59	113.47 ± 4.03	52.14 ± 17.76*	88.81 ± 4.87^†^	103.50 ± 13.82^†^	111.60 ± 10.81^†^
Creatinine clearance (mL/min)	1.85 ± 0.23	1.90 ± 0.35	1.07 ± 0.03*	1.35 ± 0.12	1.58 ± 0.05^†^	1.51 ± 0.04
U.Protein (g/dL)	0.137 ± 0.008	0.129 ± 0.021	0.196 ± 0.019*	0.172 ± 0.012	0.148 ± 0.010	0.144 ± 0.015
24-h U.Volume (mL/day)	28.8 ± 2.58	27.4 ± 3.31	13.2 ± 1.66*	18.2 ± 2.06*	16.0 ± 2.07*	21.8 ± 4.09^†^
Kidney weight (g)	1.37 ± 0.04	1.36 ± 0.01	1.52 ± 0.06*	1.39 ± 0.02^†^	1.28 ± 0.02^†,‡^	1.36 ± 0.02^†^
Kidney weight/Body weight ratio	2.75 ± 0.05	2.85 ± 0.04	3.19 ± 0.43	2.35 ± 0.03^†^	2.36 ± 0.13^†^	2.54 ± 0.03^†^
Visceral fat (g)	10.76 ± 2.73	8.69 ± 1.11	24.22 ± 3.27*	19.93 ± 0.37*^,†^	16.47 ± 2.06*^,†^	19.32 ± 0.86*

Data are presented as mean ± standard error of mean (SEM, n = 5 rats/group) ND, normal diet rats treated with vehicle; ND100, normal diet rats treated with peat moss extract 100 mg/kg/day; P-DM, pre-diabetic rats treated with vehicle. P-DM50, pre-diabetic rats treated with peat moss extract 50 mg/kg/day; P-DM100, pre-diabetic diet rats treated with peat moss extract 100 mg/kg/day; MET, pre-diabetic diet rats treated with metformin 100 mg/kg/day. Values with different superscript letters *p < 0.05 vs. ND, and ND100; ^†^ *p* <0.05 vs. P-DM; ^‡^ *p* <0.05 vs. P-DM50 (LSD, test, *p* < 0.05).

### Effects of SC extract on renal oxidative stress

**FIGURE 2:**
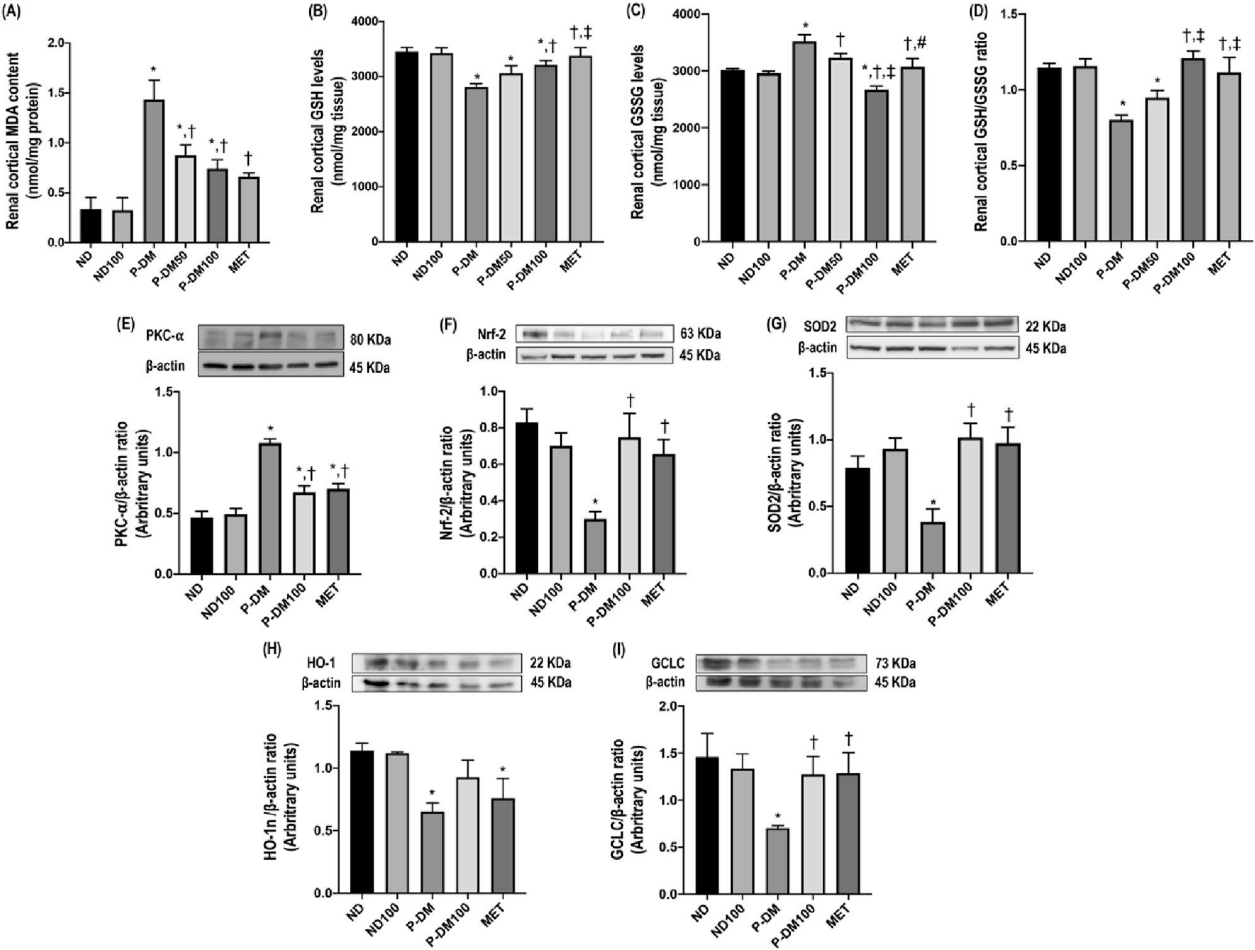
Effect of SC extract on renal oxidative stress. Renal cortical MDA content **(A)**, renal cortical GSH levels **(B)**, renal cortical GSSG levels **(C)**, renal cortical GSH/GSSG ratio **(D)**, renal cortical expression of PKC-α **(E)**, Nrf-2 **(F)**, SOD2 **(G)**, HO-1 **(H)**, and GCLC **(I)**. ND, normal diet rats treated with vehicle; ND100, normal diet rats treated with peat moss extract 100 mg/kg/day; P-DM, pre-diabetic rats treated with vehicle; P-DM50, pre-diabetic rats treated with peat moss extract 50 mg/kg/day; P-DM100, pre-diabetic diet rats treated with peat moss extract 100 mg/kg/day; MET, pre-diabetic diet rats treated with metformin 100 mg/kg/day. Values with different superscript letters *p < 0.05 vs. ND and ND100; ^†^ *p* < 0.05 vs. P-DM; ^‡^ *p* < 0.05 vs. P-DM50 indicate significant differences between treatments (LSD test, *p* < 0.05).

Malondialdehyde (MDA) is a byproduct of lipid peroxidation, serving as a biomarker for oxidative stress in obese rats subjected to a high-fat diet. The MDA in our P-DM group exhibited a statistically significant increase in comparison to both the ND and ND100 groups (*p* < 0.05) (Figure 2A). Following treatment with SC extract, the concentrations of MDA exhibited a statistically significant reduction in both the P-DM50 and P-DM100 groups (*p* < 0.05). Renal cortical level of antioxidant GSH was significantly lower in the P-DM group compared to the ND and ND100 groups (*p* < 0.05) (Figure 2B). In contrast, the GSSG level was substantially increased in the P-DM group compared to the ND and ND100 groups (*p* < 0.05) (Figure 2C). The GSH level increased and GSSG levels decreased in the P-DM100 group after SC treatment (*p* < 0.05) which indicated that SC have the potential to scavenge ROS and reduce oxidative stress. The ratio of GSH/GSSG in the P-DM condition exhibited a significant decrease compared to the ND and ND100 groups (*p* < 0.05). Interestingly, these parameters were reversed following the administration of SC therapy (Figure 2D). The findings of our study indicate that there is a disparity in the effectiveness of SC on metabolic profiles when comparing the dosage of 50 mg/kg BW to that of 100 mg/kg BW. In the next experiments, our research will concentrate on investigating the advantageous impacts of SC extract administered at a dosage of 100 mg/kg body weight in the context of obesity.

### Effects of SC extract on renal oxidative defense mechanism

A significant elevation in PKC-α protein expression was noted in the renal cortical tissue of rats subjected to a high-fat diet in comparison to rats on a normal diet that were in the ND and ND100 groups (*p* < 0.05), as depicted in Figure 2E. In contrast, the levels of Nrf2, SOD, HO1, and GCLC expression in the obese rats that did not receive any treatment were found to be considerably lower compared to the groups of rats that were given a regular diet (*p* < 0.05), as seen in Figures 2F–I. The results of this study indicate that the consumption of a high-fat diet leads to the activation of oxidative stress through the Nrf-2 pathway. The levels of Nrf-2, SOD, and GCLC expression, but not HO-1, were found to be considerably increased in the P-DM100 group compared to the P-DM group (*p* < 0.05).

### Effects of SC extract on renal morphology

**FIGURE 3:**
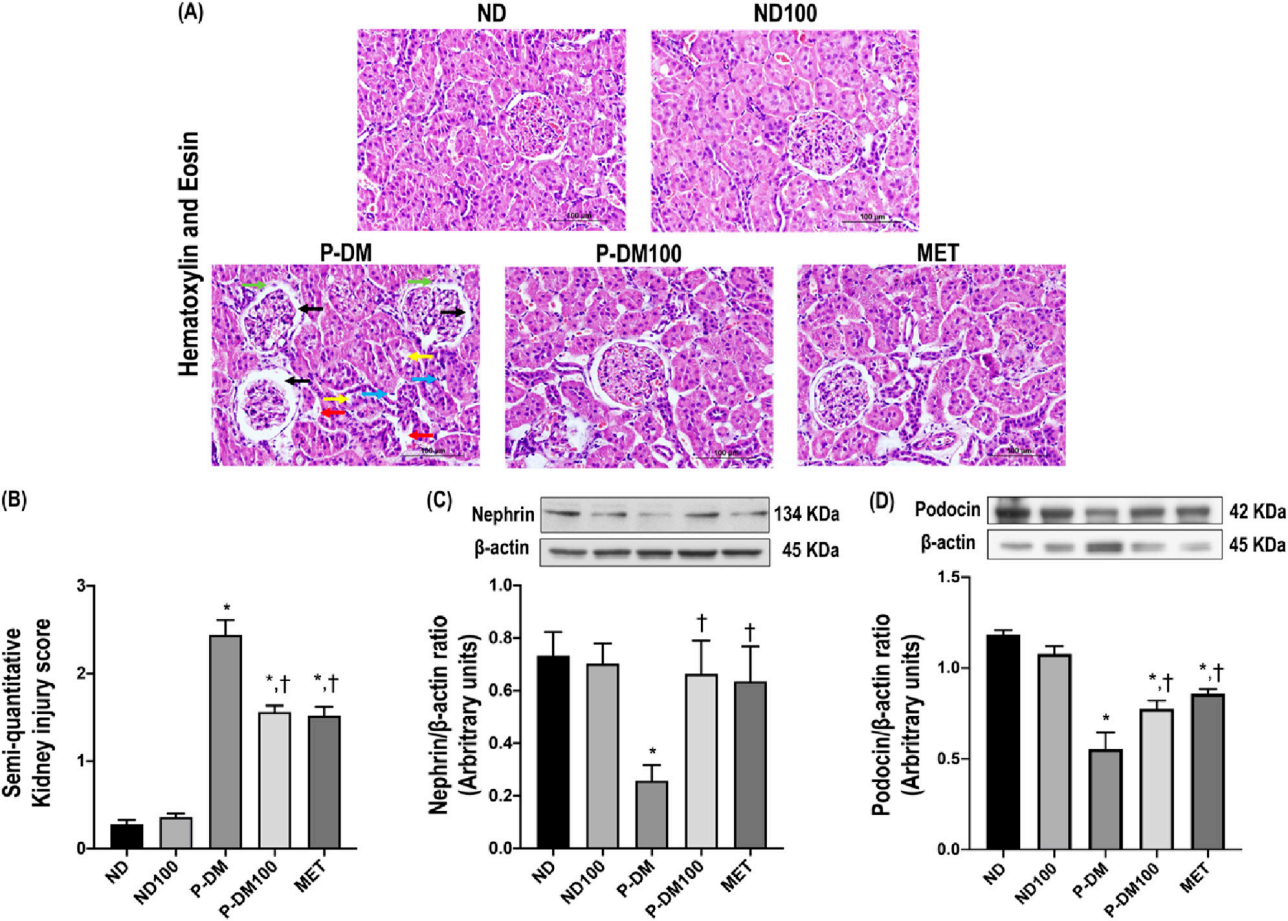
Effect of SC extract on renal morphology. Photomicrograph histological section of kidney using hematoxylin and eosin (H&E) stain (×40) in obese rats **(A)**: Dilation of Bowman’s capsule (black arrow), interstitial fibrosis (light blue arrow), macrophage infiltration (red arrow), periglomerular fibrosis (green arrow) and tubular dilation (yellow arrow), Semi-quantitative kidney injury score **(B)**, renal cortical expression of nephrin **(C)**, podocin **(D)**. ND, normal diet rats treated with vehicle; ND100, normal diet rats treated with peat moss extract 100 mg/kg/day; P-DM, pre-diabetic rats treated with vehicle; P-DM50, pre-diabetic rats treated with peat moss extract 50 mg/kg/day; P-DM100, pre-diabetic diet rats treated with peat moss extract 100 mg/kg/day; MET, pre-diabetic diet rats treated with metformin 100 mg/kg/day. Values with different superscript letters *p < 0.05 vs. ND and ND100; ^†^ *p* < 0.05 vs. P-DM; ^‡^ *p* < 0.05 vs. P-DM50 indicate significant differences between treatments (LSD test, *p* < 0.05).

The representative images of H&E staining (Figure 3) demonstrate the infiltration of macrophages, renal tubular dilation, the area of Bowman’s capsule, periglomerular fibrosis, and interstitial fibrosis. SC extract significantly attenuated renal histopathological injuries and the semi-quantitative kidney injury score in P-DM100 when compared with the P-DM group (Figures 3A, B) (*p* < 0.05). In addition, the P-DM group showed significantly reduced nephrin and podocin expression compared to the ND group (Figures 3C, D). These findings suggested that the oral administration of SC extract could improve the renal histopathological injuries induced by pre-diabetic conditions.

### Effects of SC extract on renal fibrosis

**FIGURE 4:**
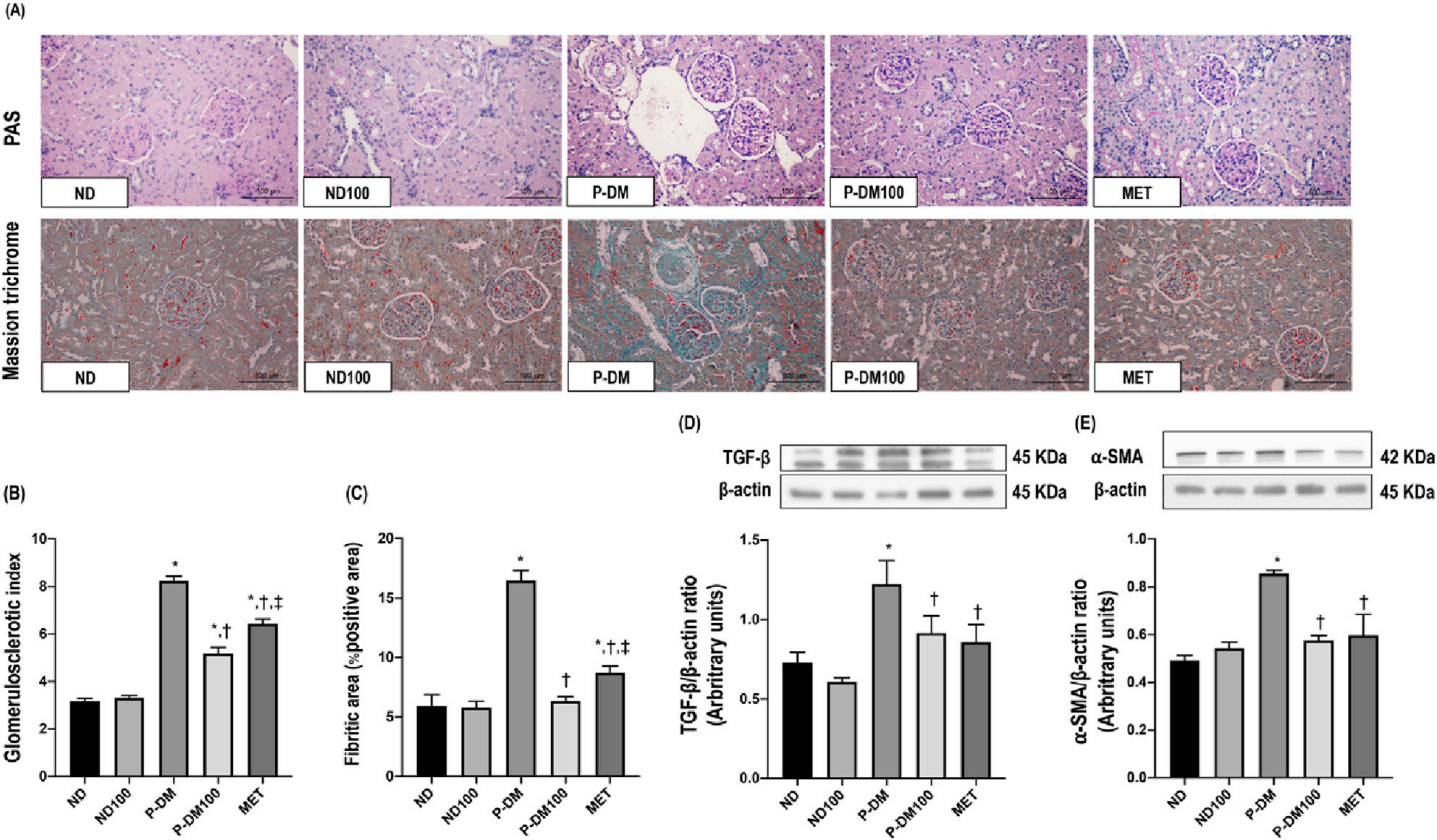
Effect of SC extract on renal fibrosis. Histological sections of kidneys stained with PAS and Masson’s trichrome (×40) **(A)**, Glomerulosclerotic index **(B)**, Fibrotic area **(C)**, Western blot analysis showing the renal expression of TGF-β **(D)**, and α-SMA **(E)**. ND, normal diet rats treated with vehicle; ND100, normal diet rats treated with peat moss extract 100 mg/kg/day; P-DM, pre-diabetic rats treated with vehicle; P-DM50, pre-diabetic rats treated with peat moss extract 50 mg/kg/day; P-DM100, pre-diabetic diet rats treated with peat moss extract 100 mg/kg/day; MET, pre-diabetic diet rats treated with metformin 100 mg/kg/day. Values with different superscript letters *p < 0.05 vs. ND and ND100; ^†^ *p* < 0.05 vs. P-DM; ^‡^ *p* < 0.05 vs. P-DM50 indicate significant differences between treatments (LSD test, *p* < 0.05).

The kidney sections taken from P-DM rats had significantly elevated levels of glomerulosclerosis, as proven by the presence of collagenous connective tissue deposits in both the glomerular and tubular regions, as observed by staining with PAS and Masson’s trichrome. This observation contrasted with the kidney sections collected from ND rats (Figure 4A). The assessment of glomerulosclerotic index and fibrotic area were significantly increased in the P-DM group compared to the ND group (*p* < 0.05) (Figures 4B, C). In addition, the P-DM rats showed significant increases in the expression of TGF-β and α-SMA in comparison to the ND rats (*p* < 0.05) (Figures 4D, E). Rats in the P-DM100 group had a significantly lower glomerulosclerotic index and fibrotic area, together with reduced TGF-β and α-SMA expression, than HF rats (*p* < 0.05).

### Effects of SC extract on renal apoptotic pathway

**FIGURE 5:**
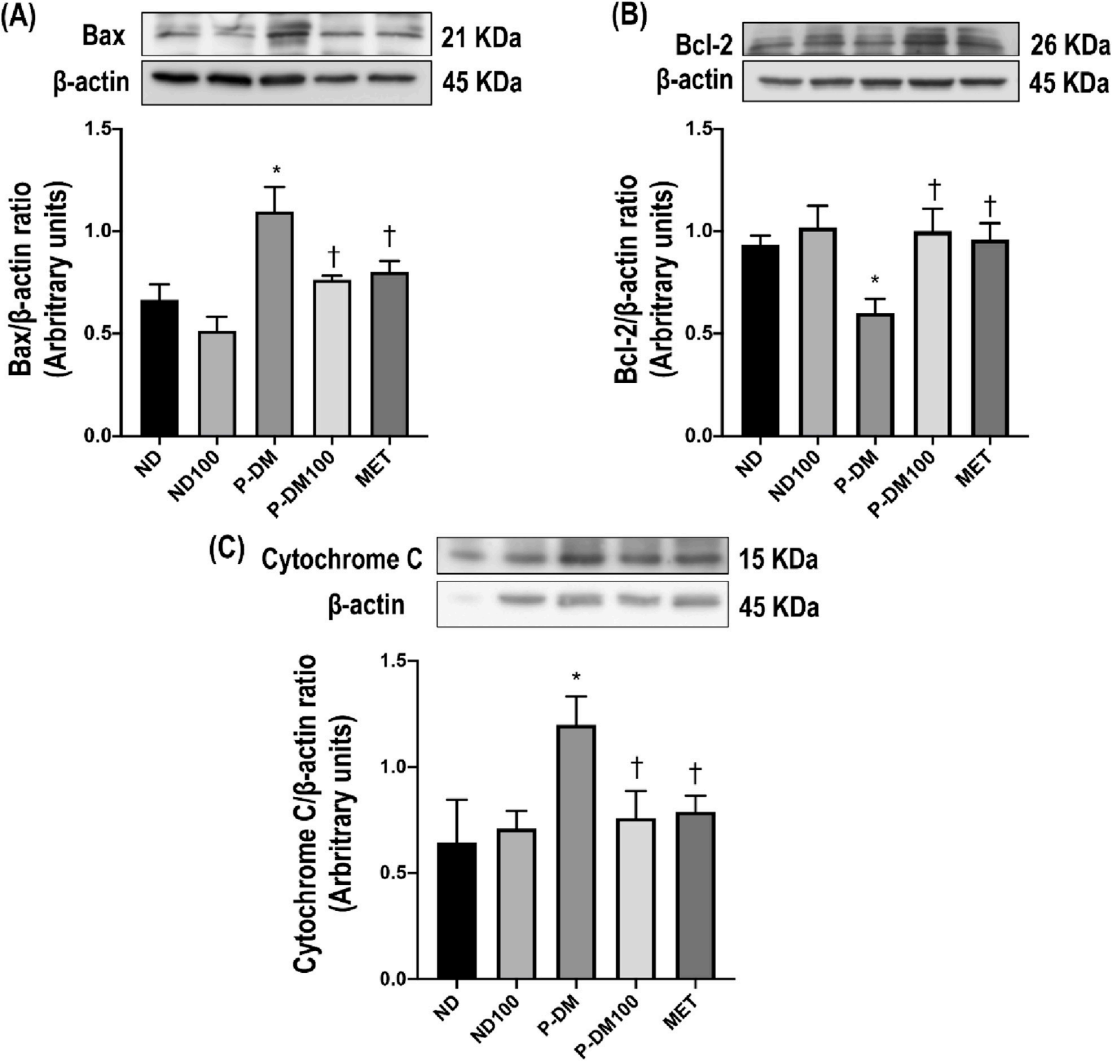
Effect of SC extract on renal apoptosis. Renal cortical expression of Bax **(A)**, Bcl-2 **(B)**, and Cytochrome C **(C)**. ND, normal diet rats treated with vehicle; ND100, normal diet rats treated with peat moss extract 100 mg/kg/day; P-DM, pre-diabetic rats treated with vehicle; P-DM50, pre-diabetic rats treated with peat moss extract 50 mg/kg/day; P-DM100, pre-diabetic diet rats treated with peat moss extract 100 mg/kg/day; MET, pre-diabetic diet rats treated with metformin 100 mg/kg/day. Values with different superscript letters *p < 0.05 vs. ND and ND100; ^†^ *p* < 0.05 vs. P-DM; ^‡^ *p* < 0.05 vs. P-DM50 indicate significant differences between treatments (LSD test, *p* < 0.05).

As shown in Figures 5A–C, high-fat diet consumption significantly increased Bax and cytochrome C expressions and markedly reduced Bcl-2 expression in renal tissues in P-DM when compared to the ND group (*p* < 0.05). SC treatment significantly downregulated the expression of Bax and cytochrome C while upregulating the expression of Bcl-2 in comparison with P-DM rats (*p* < 0.05).

## Discussion

To our knowledge, this is the first study to suggest that peat moss (*Sphagnum cuspidatulum*) extract mitigates renal impairment induced by pre-diabetes via antioxidative and anti-apoptotic capabilities. The traditional use of SC extract as a remedy for various ailments has been observed in multiple Asian countries, including Thailand. SC is a semi-aquatic moss that can be found in Aug Ka Doi Inthanon, Chiang Mai, Thailand. It contains plenty of caffeine, flavonoids, and phenolic compounds. Caffeine is a phenolic phytochemical that acts as a powerful natural antioxidant, offering various beneficial effects for humans.

This study demonstrated that the administration of a high-fat meal to rats, along with a low dosage of STZ injection, leads to several detrimental effects. These effects include increased body weight and visceral fat weight, impaired glucose tolerance, dyslipidemia, heightened oxidative stress, renal cell apoptosis, and renal dysfunction. Impaired glucose and fat metabolism elicits oxidative stress and the suppression of the oxidative defense mechanism via the Nrf-2 pathway. This imbalance between oxidant and antioxidant machinery, in turn, contributes to the development of renal tissue damage. In addition, pre-diabetic conditions might lead to renal cell death by upregulating the apoptotic proteins Bax and cytochrome C while simultaneously downregulating the expression of the anti-apoptotic protein Bcl-2.

The administration of phenolic compounds to rats subjected to a high-fat diet leads to a decrease in plasma glucose levels and an improvement in lipid metabolism (Hanhineva et al., 2010). The results from our study also demonstrate that phenolic compounds obtained from SC extract effectively reduce plasma glucose levels in P-DM rats.

The visceral fat accumulates around the kidneys in obese pre-diabetic rats. The physical compression of the kidney via perirenal adipose mass may reduce renal tubular flow in the distensible loop of Henle, which leads to an increase in glomerular filtration rate (GFR) (Kataoka et al., 2023). Similar to a previous study, this study also demonstrates that the accumulation of visceral fat is associated with elevated GFR and blood creatinine levels (Gerchman et al., 2009). Nephrin and podocin are important proteins of the glomerular filtration barrier. In our study, P-DM rats had decreased expression of renal cortical nephrin and podocin, which was reversed by the administration of SC extract.

Hyperlipidemia contributes to the gradual decline of renal function as a result of oxidative stress, endoplasmic reticulum stress, inflammatory reactions, and the restricted proliferative capacity of glomerular and mesangial cells (Chen et al., 2023). In this study, we observed the histological changes in the renal tissues of pre-diabetic rats, including macrophage infiltration, dilatation of Bowman’s capsule, and fibrosis in both the glomerular and tubular regions. TGFβ and αSMA are the important proteins involved in the processes of glomerulosclerosis and tubulointerstitial fibrosis, which are the common pathological features of kidney diseases. We observed that SC extract reduced the expression of TGFβ and αSMA in the P-DM rats. Our study was confirmed by another experiment, caffeine can activate AMPK and SIRT3 that inhibits several fibrogenic pathways (Xu et al., 2020), including those mediated by TGF-β, a key player in kidney fibrosis can cause partial epithelial to EMT and EndMT. This inhibition can reduce the production of fibrotic extracellular matrix components (Srivastava et al., 2021b).

The presence of heightened oxidative stress has a pivotal role in the development of nephropathy in individuals with pre-diabetes (Vodošek Hojs et al., 2020). A chronic state of hyperglycemia augments the generation of ROS, inducing oxidative stress (González et al., 2023). The elevation in MDA levels, which is regarded as a marker of oxidative stress (Jaikumkao et al., 2018; Swe et al., 2019), seen in pre-diabetic rats in our study. To mitigate the effects of oxidative stress, cells employ a detoxification mechanism, which includes the Nrf-2 pathway. This system serves as a key regulator of cytoprotective responses in the face of oxidative stress (Ngo and Duennwald, 2022). Under physiological circumstances, Nrf-2 is typically confined to the cytosol, where it forms a complex with the Kelch-like ECH-associated protein 1 (Keap1). The translocation of Nrf-2 to the nucleus is induced by the ubiquitination and proteasomal degradation of Keap1 in the presence of ROS. This translocation facilitates the translation of enzymes associated with antioxidant activity (Baird et al., 2020) such as SOD, GCLC, HO-1 and, GSH. The GSH and GSSG exhibit exceptional sensitivity to variations in ROS flux caused by alterations in the glutathione reservoir in reaction to elevated levels of oxidative stress (Mailloux and Treberg, 2016). In our study, renal cortical Nrf-2 levels decreased which may be due to long-term oxidative stress from pre-diabetic conditions (Figure 4D). This result was supported by previous studies in which Nrf2 expression was reduced in obese condition (Cheng et al., 2011; Tan et al., 2011).

**FIGURE 6:**
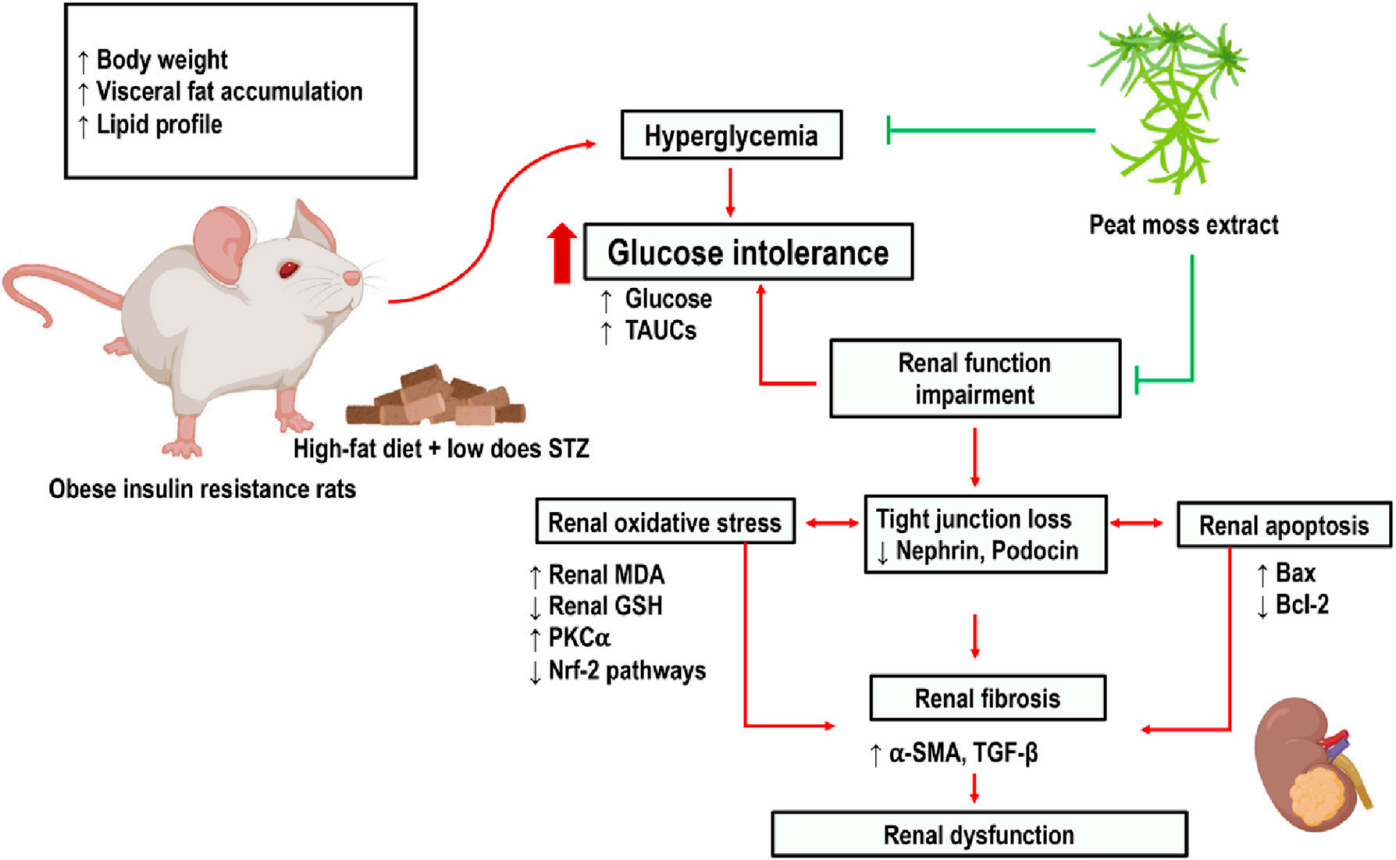
The pathway figure summarizing the findings of the study.

SC have high level of caffein can enhance the expression of the catalytic subunit of α-glutamyl cysteine synthase, which governs the rate at which glutathione is produced (Myhrstad et al., 2002). According to our research, the phenolic compounds obtained from the SC extract exhibit a stimulatory impact on the concentration of glutathione while simultaneously decreases the levels of MDA in the renal tissue. Furthermore, our investigation reveals that the administration of SC extract increases the expression of Nrf-2 and its associated signaling molecules, such as GCLC, HO-1, SOD, and GSH as a defensive mechanism in response to renal oxidative injury (Figure 6).

In our previous study, we found that a high-fat diet induced renal injury via oxidative stress and apoptosis (Laorodphun et al., 2021). The present study also provides evidence for the activation of the apoptotic pathway in P-DM rats. The expression of the pro-apoptotic protein Bax is increased, which is accompanied by a decrease in the levels of the anti-apoptotic protein Bcl-2. Furthermore, this apoptotic activation is shown to be related to an upregulation of cytochrome C. SC extract can reverse these changes. Various studies have demonstrated the diverse health benefits attributed to phenolic compounds, encompassing their antioxidative, antihyperglycemic, antihyperlipidemic, and anti-inflammatory capabilities (El-Hadary and Ramadan, 2019; Singh and Yadav, 2022). In our study, we demonstrated that SC extract also possesses anti-apoptotic effects. In conclusion, our investigation has revealed that the SC extract has various therapeutic effects in a pre-diabetic rat model. The mechanisms of the beneficial effects of SC extract include the mitigation of hyperglycemia, hyperlipidemia, oxidative stress, apoptosis, and renal injury.

## Conclusion

Our study shows the potential of SC extract in alleviating hyperglycemia, dyslipidemia, renal morphological changes, and renal dysfunction in pre-diabetic rats induced with a high-fat diet and streptozotocin. We demonstrate that the renoprotective effect of SC extract is due in part to the antioxidant and anti-apoptotic properties of the extract. However, further clinical trials in humans are required to provide important details about the efficacy and safety before use of SC extract as an alternative treatment for renoprotection in patients with diabetes.

### Study limitation

Our study is limited to the precise exploration of how the kidney responds to ROS signaling, a crucial regulator of renal function. We would like to address the limitation related to the study of renal cortical cell lysates as recent research has revealed that the kidney is composed of over 20 distinct cell groups, many of which exhibit gradual variations in their gene expression patterns, as determined by single-cell and single-nucleus RNA sequencing techniques. Future research should focus on exploring the intricate variations in gene expression patterns across distinct renal cell types.

## Data Availability

The original contributions presented in the study are included in the article/supplementary material, further inquiries can be directed to the corresponding author.
